# A preclinical radiotherapy dosimetry audit using a realistic 3D printed murine phantom

**DOI:** 10.1038/s41598-022-10895-5

**Published:** 2022-04-26

**Authors:** Emma R. Biglin, Adam H. Aitkenhead, Gareth J. Price, Amy L. Chadwick, Elham Santina, Kaye J. Williams, Karen J. Kirkby

**Affiliations:** 1 Division of Cancer Sciences, Faculty of Biology, Medicine and Health, The University of Manchester, 3rd floor Proton Beam Therapy Centre, Oak Road, Manchester, M20 4BX UK; 2 Christie Medical Physics and Engineering, The Christie NHS Foundation Trust, Manchester, UK; 3 The Christie NHS Foundation Trust, Manchester, UK; 4 Division of Pharmacy and Optometry, Faculty of Biology, Medicine and Health, University of Manchester, Manchester, UK

**Keywords:** Physics, Translational research, Cancer

## Abstract

Preclinical radiation research lacks standardized dosimetry procedures that provide traceability to a primary standard. Consequently, ensuring accuracy and reproducibility between studies is challenging. Using 3D printed murine phantoms we undertook a dosimetry audit of Xstrahl Small Animal Radiation Research Platforms (SARRPs) installed at 7 UK centres. The geometrically realistic phantom accommodated alanine pellets and Gafchromic EBT3 film for simultaneous measurement of the dose delivered and the dose distribution within a 2D plane, respectively. Two irradiation scenarios were developed: (1) a 10 × 10 mm^2^ static field targeting the pelvis, and (2) a 5 × 5 mm^2^ 90° arc targeting the brain. For static fields, the absolute difference between the planned dose and alanine measurement across all centres was 4.1 ± 4.3% (mean ± standard deviation), with an overall range of − 2.3 to 10.5%. For arc fields, the difference was − 1.2% ± 6.1%, with a range of − 13.1 to 7.7%. EBT3 dose measurements were greater than alanine by 2.0 ± 2.5% and 3.5 ± 6.0% (mean ± standard deviation) for the static and arc fields, respectively. 2D dose distributions showed discrepancies to the planned dose at the field edges. The audit demonstrates that further work on preclinical radiotherapy quality assurance processes is merited.

## Introduction

The late 2000s saw the first application of sophisticated small animal irradiation platforms to deliver complex preclinical irradiations and implement image guidance^1,2^. Since then, as these machines became more widespread and regularly implemented, considerable technological advances have been made in the imaging, planning and delivery of preclinical irradiation, to keep up with the fast-paced advances of their clinical counterparts^3^. Despite recent improvement, preclinical irradiation workflows still do not mirror the standardized and rigorous commissioning procedures, dosimetry quality assurance (QA) checks and national auditing procedures for clinical radiation systems^4–8^.

There are two main types of irradiator commissioning: equipment commissioning^9–12^, and technique commissioning^13–15^. General equipment commissioning is manufacturer specific and a standardized practice across different irradiators is currently not available. It is often completed upon installation but may be repeated if dosimetry checks have been neglected for some time. General commissioning alone may not be sufficient for complex experimental designs, therefore the commissioning of individual techniques is vital for accurate irradiations. In terms of QA, there are numerous different approaches employed by the small animal radiation research community in terms of the tests that are required ^16,17^, who should undertake these and at what frequency.

The lack of guidance and implementation of reporting vital radiation parameters hinders reproducibility and is one of the contributing factors to a paucity in dosimetry standardization^18,19^. Other in vivo research areas rely on the ARRIVE guidelines (Animal Research: Reporting of in Vivo Experiments), as set out by the NC3Rs (National Centre for the Replacement Refinement and Reduction of Animals in Research), documenting the parameters that should be reported to facilitate accurate reproducibility^20^. At present these do not include factors relating to radiation research. However, Verhaegen et al*.*^18^ have made recommendations for reporting radiation parameters which include the radiation modality, dose prescription, treatment field, target margins and optimisation parameters.

Ableitinger et al.^21^ recommend that if multiple institutions partake in collaborative studies then a dosimetry audit across the sites should be mandatory to ensure sufficient dosimetric accuracy, thereby increasing confidence in the comparability and reproducibility of the results. In the present study, we used a postal audit to investigate dosimetric conformance of UK image-guided small animal precision irradiation facilities. Measurements of the dose delivered for both static and arc beam geometries were made using a realistic murine phantom containing Gafchromic EBT3 film (Vertec Scientific Ltd. Reading, UK) and alanine dosimeters (NPL, Middlesex, UK), and were compared against the dose predicted by the treatment planning systems (TPS). We additionally surveyed participating institutes to ascertain the equipment and techniques currently supported by departments, quality control processes in use, and attitudes to the need for quality control in pre-clinical radiation experiments.

## Methods

### Questionnaire

Prior to the audit, information regarding the equipment in use, the techniques implemented and the QA procedures in place at 7 centres across the UK which actively undertake in vivo radiation research using an Xstrahl (Walsall, UK) Small Animal Radiation Research Platform (SARRP) was gathered via a questionnaire. The questionnaire also invited participants to give their opinion, through open-ended questions, on the necessity of dosimetry audits and defined protocols, the level of acceptable dose tolerances, QA responsibilities and required reporting parameters in publications to improve the currently poor reproducibility of research^18^.

### Phantom design and printing

**Figure 1:**
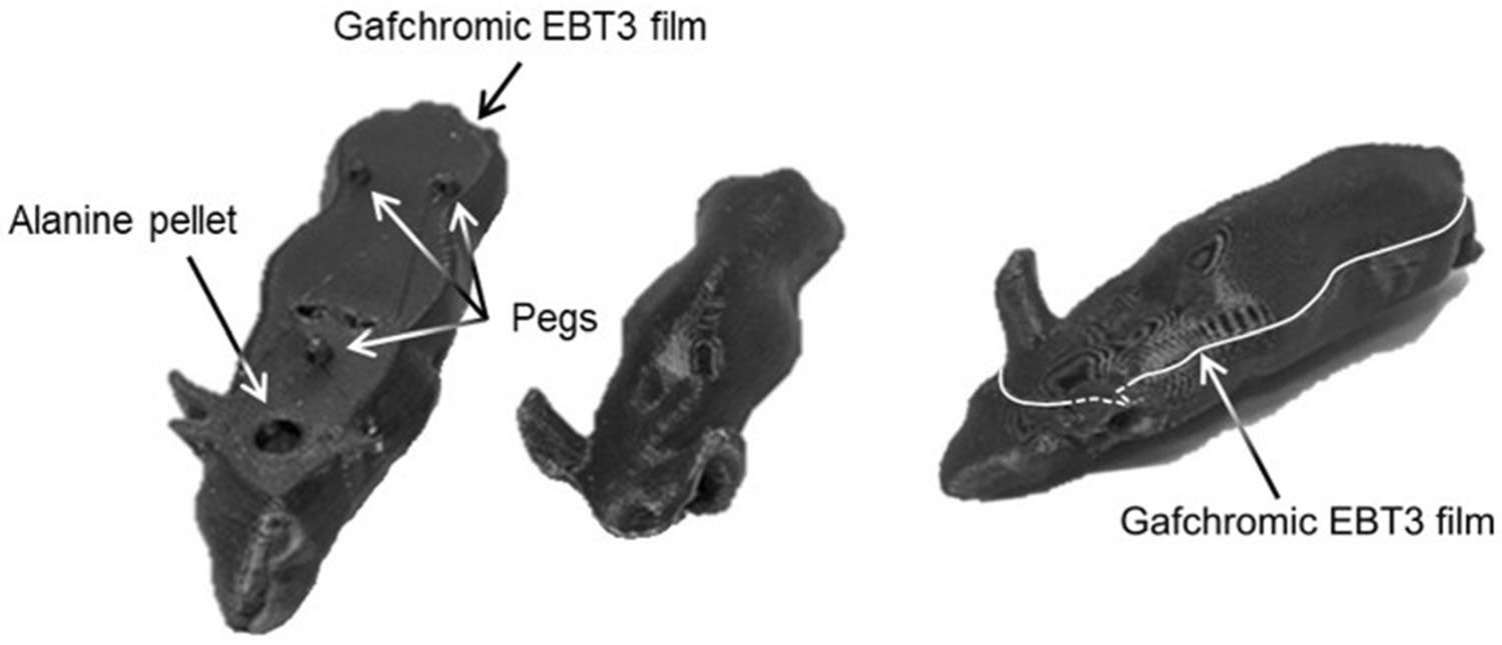
The 3D printed murine dosimetry phantom. An Ultimaker 3 fused deposition modelling printer was used to create bespoke phantoms capable of securing Gafchromic EBT3 film over an alanine pellet to capture an absolute dose measurement and dose distribution with reference to a national primary standard. The assembled phantom is shown on the right. A second version of the same phantom was printed with the alanine pellet cavity situated in the pelvis region.

The phantom used in this audit was previously described by Price et al*.*^22^ (which includes a link to open source files for the phantom geometry). To create the phantom the cone beam computed tomography (CBCT) scan of a nude mouse was segmented into three parts (body, bones and lungs) then transformed into stereolithographic files suitable for import into Meshmixer (Autodesk, Inc.) and Netfabb (Autodesk, Inc.) computer-aided design software. In this study, the phantom (body and lungs) was split on the central coronal plane to accommodate Gafchromic EBT3 film, and a cylindrical cavity 6 mm in diameter and 2.5 mm in height was incorporated in the ventral half of the phantom, in the brain region, to contain an alanine detector. The use of a higher density bone-equivalent material was excluded from this study to avoid any dosimetric uncertainties relating to the tissue segmentation process within the SARRP Muriplan treatment planning system^23^. Three pegs (5 mm length, 3 mm diameter) were included in the design to hold the film in place (Fig. 1). A second phantom but with the pellet cavity located in the pelvis region was also designed. The overall dimensions of the phantom were 86.9 mm (length), 30.5 mm (width), and 26.3 mm (height).

This design allowed simultaneous irradiation of the film and alanine detectors to allow a direct comparison between measurements. All plans were designed so that the beam was incident on the dorsal surface of the phantom, passing through the film and then through the alanine pellet located directly beneath the film. Gafchromic EBT3 film is thin (< 0.3 mm) and relatively water/tissue equivalent, so is unlikely to perturb the dose distribution downstream of the film. To avoid any risk of the bulkier alanine pellets perturbing the dose to the film, the alanine pellets were located beneath the film. The use of a realistic murine phantom over a simple cylindrical geometry allows a representative test of the treatment pathway, from phantom positioning, image-guided treatment planning and treatment delivery.

The 3D printing parameters and gcode for the final phantom design were prepared using Ultimaker Cura software^24^, and the phantoms were printed using an Ultimaker 3 (Ultimaker BV, Utrecht, Netherlands) fused deposition modelling (FDM) 3D printer. The material used was polylactic acid (PLA, RS Components Ltd.) which has a density of 1.25 g/cm^3^ in raw filament form. Although the phantoms were printed at 100% in-fill, the measured density of the final printed phantoms (1.19 g/cm^3^) was lower than that of the raw material due to effects within the printing process^22^. The choice of PLA for the phantom material was made based on its availability and suitability as a tissue substitute^25,26^. The lungs were incorporated into the model as air cavities.

The geometrical fidelity of the printed phantoms was assessed in terms of the distance-to-agreement (DTA) between the surface of the phantom (imaged by CBCT) and the design (defined by STL file). The resulting distribution of DTA values was centred on zero with a standard deviation of 0.3 mm, showing that the final printed phantoms accurately replicated the design.

### Audit procedure

For logistical reasons one institution who took part in the questionnaire was unable to participate in the dosimetry audit. Two murine phantoms (for separate brain and pelvis irradiations) were delivered to the remaining six institutions, herein referred to as S1-S6. Each of these institutions had their own SARRP installation.

**Figure 2:**
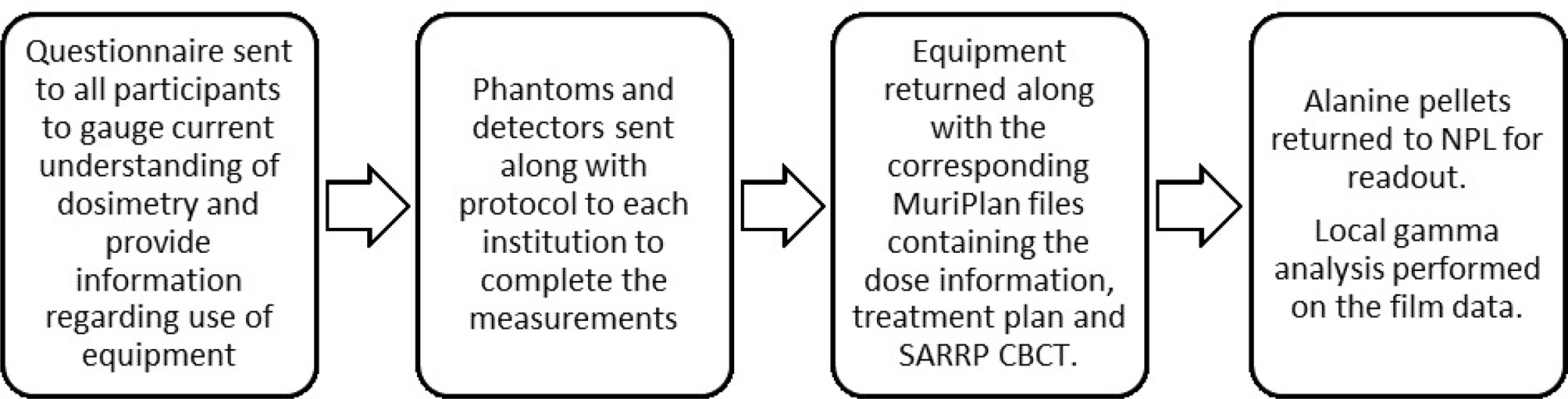
Workflow of the audit.

The audit workflow (Fig. 2) was designed to simulate the procedure of a typical in vivo experiment: CBCT acquisition, treatment planning and beam delivery.

For the end-to-end audit, 2 scenarios were assessed:

1. Delivery of a simple static field to the pelvis using a 10 × 10 mm^2^ collimator, with the bed and gantry at 0°.
2. Delivery of a more complex 90° arc field to the brain using a 5 × 5 mm^2^ collimator. The bed and gantry angles were 90° and − 45° to 45° respectively, to create an arc in the sagittal plane intended to be representative of techniques designed to spare one hemisphere of the brain. The arc plan was intentionally designed to be more challenging than the static field plan, and to test the limits of what might be attempted in a pre-clinical setting were a field size to be chosen to conform tightly to the intended target.

**Figure 3:**
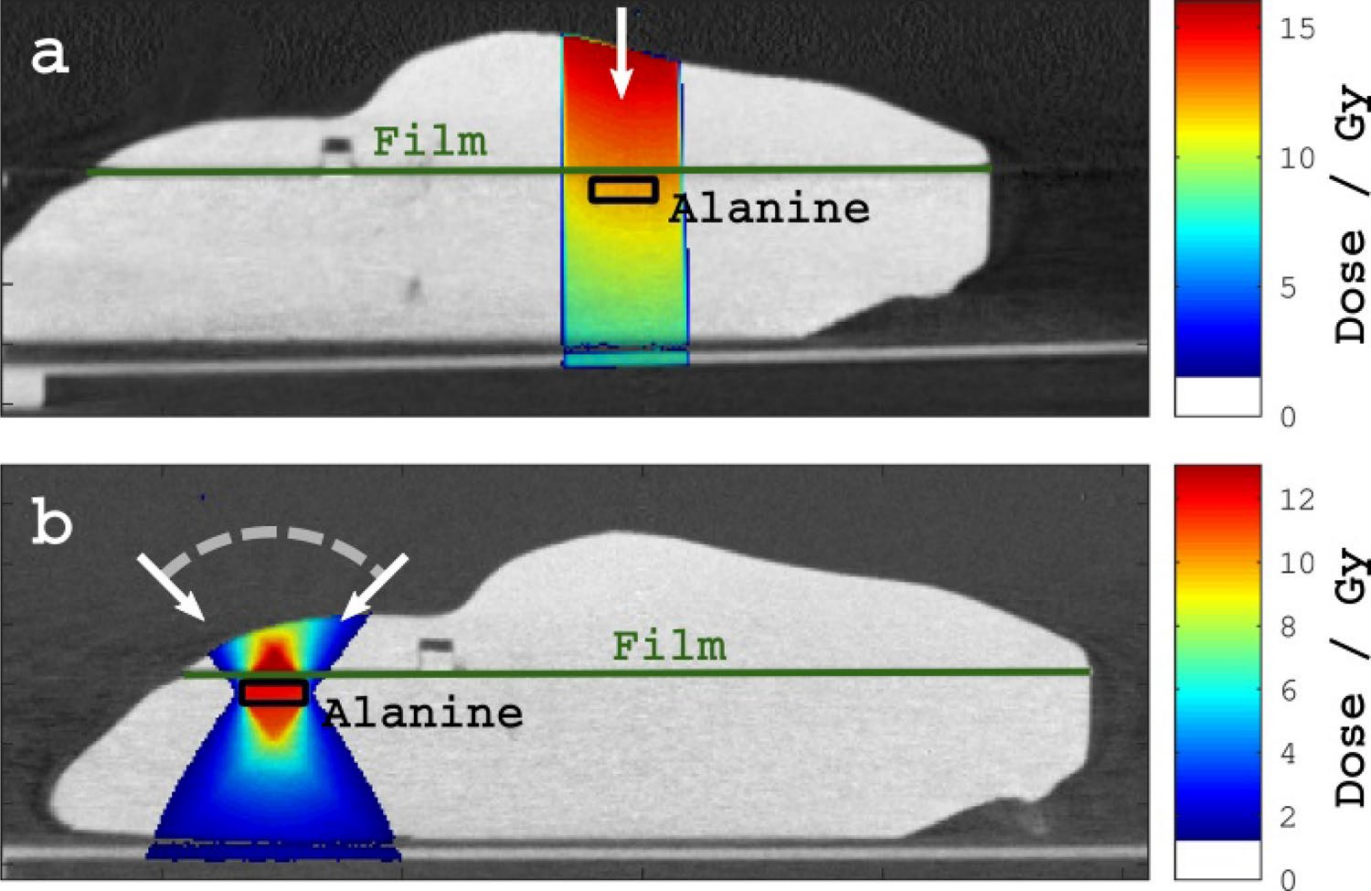
Illustration of the plan designs for (**a**) the simple irradiation of the pelvis using a static 10 × 10 mm static field, and (**b**) the more complex irradiation of the brain using a 90° arc in the sagittal plane and a 5 × 5 mm collimator. The arrows indicate the beam directions.

The plan layouts are illustrated in Fig. 3. For both scenarios the prescribed dose of X-rays was 12 Gy to the isocentre, set in the centre of the alanine detector, and the source settings were 220 kVp and 13 mA. Each scenario was planned and delivered twice, with new alanine and film, to obtain repeat measurements. The prescribed dose of 12 Gy was chosen to suit the sensitivity range of the alanine dosimeters.

Each centre was provided with a protocol documenting the procedure and equipment handling instructions (Supplementary Table S1), four pre-labelled laser cut films and alanine pellets and spares. For the SARRP irradiations, participants were instructed to follow their standard operating procedure to acquire a CBCT of the phantom with the alanine and film in place and develop a treatment plan on the resulting image using the SARRP Muriplan TPS. As per standard SARRP operating procedure, soft tissue and lung (air) were segmented using pixel intensity thresholding and the standard bulk density overrides were applied.

### Alanine dosimetry

The alanine detectors were purchased from the alanine dosimeter reference service at the National Physical Laboratory (NPL) to measure the absorbed dose to water^27^. The detectors were 5 mm in diameter and 2.3 mm in height.

After use, the alanine pellets were returned to NPL for readout, < 57 days after irradiation. The dose reported is traceable to the primary standard for Cobalt-60 beam quality and therefore a correction factor is required when used with low and medium energy X-rays due to an energy dependence^23^. This correction factor is based on each irradiator’s half-value layer (HVL), the thickness of a material (most often aluminium or copper) required to attenuate the intensity of radiation by half^28^. The HVL value was provided by each institution, either following the manufacturer’s specification (S3, 4, 5 and 6) or from their own commissioning measurements (S1 and 2). The correction factor corresponding to each HVL value was calculated using the formula, described by Silvestre Patallo et al.^23^ based on the correction factors measured in the NPL’s medium energy reference beam using HVL values between 0.5 and 4 mm Cu. The resulting absorbed doses were compared to the median planned dose to the pellet volume and the percentage difference calculated.

To determine the median planned dose to the pellet volume, the CBCT image and dose grid were first exported from the TPS. Both datasets were imported into an Octave environment^29^ for analysis, where the centre of the alanine pellet was identified within the CBCT image. A volume of diameter 5 mm and height 2.3 mm (matching the dimensions of the alanine pellet) was generated centred on this location, and was mapped onto the dose grid. All voxels within this volume were identified, with the median value being used as the planned dose in the analysis.

### Gafchromic EBT3 film dosimetry

A set of calibration films was irradiated using the 300 kV source at the NPL to allow a calibration curve to be generated for the batch of Gafchromic EBT3 film used in the audit. Irradiations were delivered using a beam quality of 0.5 mm Cu HVL, source-to-surface (SSD) distance of 75 cm with solid-water (WT1) slabs arranged to provide 2 cm depth and 20 cm backscatter. 5 × 4 cm sections of film were irradiated at doses of 0, 1, 2, 3, 4, 5, 6, 9, 12 and 15 Gy to create the calibration reference.

All films (calibration and measurements) were scanned using an Epson 10000XL flatbed scanner in transmission RGB mode, colour corrections disabled, at a spatial resolution of 400 dpi and a bit depth of 16-bits per colour channel. A minimum time between irradiation and scanning of 24 h was left to allow the polymerisation of the active layer within the film to stabilise^9,30^. Scanned images were stored in TIFF format and all subsequent analyses were done using the original spatial resolution of 400 dpi. All images acquired using the Epson 10000XL scanner were pre-processed prior to use to correct for scanner non-uniformity issues. The correction factor at each pixel of a scanned image is dependent on three things: (1) the colour channel; (2) the position of the pixel on the scanner bed (in terms of the distance from the central axis of the scanner); (3) the darkness of the film at that point. A correction map was previously created using a series of films, uniformly irradiated at doses from 0 to 25 Gy, and scanned at all positions across the scanner bed. This allowed a correction map to be created as a function of colour channel, position and optical density.

**Figure 4:**
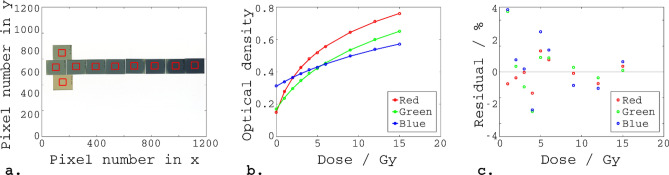
Calibration of the Gafchromic EBT3 film. (**a**) The calibration films. For each film, the median optical density within the region-of-interest marked by the red box was used for creation of the calibration curves. (**b**) The calibration curves, relating optical density and dose for each colour channel. The circles represent the median measured optical densities, and the lines represent the fits of the form: Dose = A × B^O.D.^ + C. (**c**) The residuals for each colour channel.

The calibration curve was parametrised using an equation of the form shown in Eq. (1), as described by Aitkenhead et al.^31^.

1 $${\text{Dose}} = {\text{A}} \times {\text{B}}^{{{\text{O}}.{\text{D}}.}} + {\text{C}}$$

where A, B, C are the fit parameters and O.D. represents the optical density. Figure 4 shows the calibration films, the fits and residual differences between the fits and the delivered doses for each colour channel.

To fit the exact geometry of the 3D printed design, sheets of Gafchromic EBT3 film were laser cut to form the outline of the phantom and accommodate the pegs. To generate the dimensions for the laser cutting, Netfabb was used to convert the model into slices and the slice corresponding to the film location was exported as a DXF file, compatible with the laser cutting software. The asymmetric geometry of the film aided both the fitting of the film into the phantom in the correct orientation, and locating the position of the film in the treatment plan during its analysis.

Film measurements of the delivered dose were compared to that calculated by the TPS using gamma analysis^32^. Analysis was performed within an Octave environment^29^ using in-house software which has previously been clinically commissioned for other applications^31,33,34^. For each centre, the film data formed the reference dataset while the planned dose grid formed the evaluation dataset, following the terminology used by Low et al.^35^. The same 3D planned dose grid exported from the TPS as part of the alanine analysis was as the evaluation dataset. The software performed a full 3D gamma analysis for the reasons described by Pulliam et al.^36^, calculating the gamma index for each pixel in the 2D reference image by minimising the dose difference (DD) and DTA within the 3D evaluation image.

The film analysis procedure was designed to allow separate evaluation of (1) the positional agreement between the planned and delivered dose, (2) the absolute dose delivered and (3) the shape of the dose distribution:

1. For each film, the outline geometry and 3 holes corresponding to the pegs shown in Fig. 1 were used to geometrically locate the film within the phantom. The position of the film was then manually adjusted by applying small offsets to best match the field edges to the planned dose distribution. The positional offset applied was recorded as the residual shift.
2. The reference (film) data was normalised to the evaluation (plan) data prior to the gamma analysis in order to assess differences in the dose distribution shape without results being dominated by any difference in absolute dose. Normalisation factors were calculated for all pixels in the film within the 90% isodose region (relative to the prescribed dose of 12 Gy) relative to the corresponding point in the planned dose grid. The median of these factors was taken as the overall normalisation factor for that film. We chose to use the 90% isodose area for normalisation in order to include only the high dose region, excluding the penumbral region where agreement between film and plan is less likely to be reliable.
3. The shape of the dose distribution was evaluated by performing gamma analyses for a range of DD criteria from 2 to 7%, and a DTA criterion of 0.3 mm. Dose differences were evaluated relative to the local dose. All pixels in the film corresponding to a dose > 4% of the prescribed dose (12 Gy) were evaluated within the analysis.

The normalisation factor described above also provided a means to evaluate the agreement between the film and plan in terms of absolute dose. A normalisation factor equal to 1 would imply that the film measurement agreed in terms of absolute dose with the TPS (on average, over the area within the 90% isodose line), while a normalisation factor greater than 1 would correspond to the TPS dose being greater than the film. This method of evaluating the absolute dose over an area is more reliable than evaluating the dose to a single point, as it is insensitive to noise in the film measurement (Note that we use the term *absolute dosimetry* to refer to measurement of the magnitude of the delivered dose, to distinguish from the measurement of the shape of the dose distribution).

## Results

### Questionnaire feedback

A summary of the questionnaire results can be found as Supplementary Table 2 online. All institutions agreed that audits and defined dosimetry protocols are important. Most participants suggested that a dose tolerance of within 5% was an acceptable dose agreement and one centre proposed that < 15% would be satisfactory. Variations between centres arose with the questions pertaining to QA tests. Using various ionisation chambers these were either completed every 6 months (in two centres by the manufacturer), every 2 months, whenever the machine was used or daily. The calibration of three of these farmer-type ionisation chambers could be traced to the national primary standard and another two centres’ chambers were calibrated by the chamber manufacturer (PTW-Freiburg GmbH). Output checks were performed either annually, bi-annually, monthly or bi-monthly and the output was calibrated at least every 2 years.

With regards to reporting parameters, each institution gave the following responses:

- S1—No response
- S2—Device used, gating used, dose delivered, dose rate, fractionation, image guidance, irradiation technique, field size, SSD, backscatter, couch position, D95, HVL, voltage, filtration, dosimetry protocol (air or water), output measurements, depth, backscatter, target medium, calibration conditions.
- S3—No response
- S4—Dose, isocentre, isolines, organ at risk sparing,
- S5—The dose delivered and the dose distribution,
- S6—Dose, dose rate, irradiation protocol, geometry, collimation, commissioning, tube current, filtration and the dose received by 90% of the target (D90) and
- S7—Filters, setup, energy, mAs, dosimetry equipment, field size, beam quality.

Although the suggestions varied widely, all participants agreed that more informed reporting would lead to better reproducibility of research.

### Alanine dosimetry

The HVL thickness of the individual irradiators can be used to account for the difference in response of alanine between medium energy X-ray and Cobalt-60 beams^23^. The HVL values reported for all institutions ranged from 0.65 mm Cu to 0.85 mm Cu corresponding to energy dependence factors of 0.79–0.81, respectively (Supplementary Table S3).

**Figure 5:**
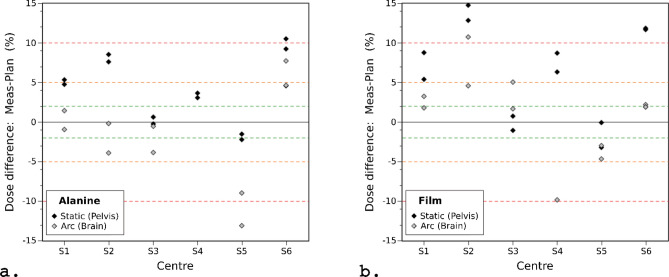
Percentage difference between planned and measured doses on the SARRP irradiators for the (**a**) alanine and (**b**) film measurements. A positive value indicates the measured dose was greater than the planned dose. The dashed green, orange and red lines indicate 2%, 5% and 10% differences from the prescribed dose, respectively.

The static beam alanine measurement, in which the 10 × 10 mm^2^ field covered the whole alanine pellet in the plan (5 mm diameter), was used as an accurate determinant of the delivered dose. The results, after applying the energy dependence correction factors, are presented in Fig. 5a. All but 1 measurement achieved < 10% deviation from the planned dose, and more than half of the measurements were within ± 5%. The mean absolute difference was 4.8%. The difference between the planned dose and alanine measurement across all centres was 4.1 ± 4.3% (mean ± standard deviation), with an overall range of − 2.3 to 10.5%.

Alanine irradiation in the phantom using the more complex beam configuration of an arc (Fig. 5a) resulted in lower measured doses than for the static beam. All but 1 measurement achieved < 10% deviation from the planned dose, and more than half of the measurements were within ± 5%. The mean absolute difference was 4.5%. The difference between the planned dose and alanine measurement across all centres was -1.2% ± 6.1% (mean ± standard deviation), with an overall range of − 13.1 to 7.7%.

For each individual SARRP, the dose difference between the repeat measurements ranged from 0.1 to 1.0% for the static beam, and from 2.0 to 3.7% for the arc delivery.

A summary of the alanine dosimetry results can be found in Supplementary Table S3.

### Gafchromic EBT3 film dosimetry

The residual shifts required to match the film position to the plan were consistent across all centres, with an average residual of 0.68 ± 0.35 mm (mean ± standard deviation) across all centres and deliveries. These residuals may represent the total of all sources of error, including delivery issues such as the difference between the true isocentre position and the isocentre position modelled in the TPS, as well as measurement issues such as the accuracy of positioning the film within the phantom.

For Gafchromic EBT3 film, the absolute dose measurements were derived from the normalisation factors computed during the gamma analysis procedure (described earlier), and the results are presented in Fig. 5b. 79% of measurements achieved < 10% deviation from the planned dose, and more than half of the measurements were within ± 5%. The mean absolute difference from the planned dose was 6.7% and 4.2% for the static and arc deliveries, respectively. The difference between the planned dose and film measurement across all centres was 6.0 ± 6.2% (mean ± standard deviation) with an overall range of − 3.2 to 14.7% for the static deliveries, and 1.3 ± 5.2% (mean ± standard deviation) with an overall range of − 9.9 to 10.7% for the arc deliveries.

**Figure 6:**
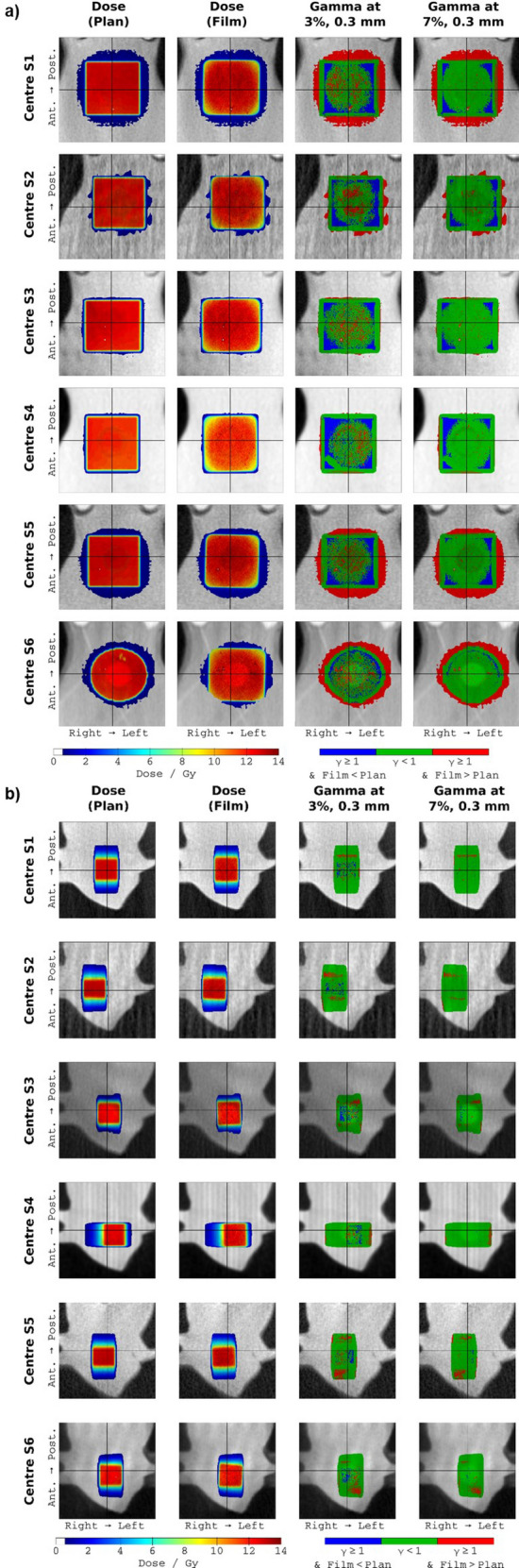
Results of the gamma analyses. (**a**) Results of the gamma analyses for one film from each centre for the static plan. Each row presents results for one centre (S1–S6). The columns present the planned dose, the film dose, and the gamma results at 3%, 0.3 mm and 7%, 0.3 mm, respectively. Each image has dimensions of 20 × 20 mm. (**b**) Results of the gamma analyses for one film from each centre for the arc plan. Each row presents results for one centre (S1–S6). The columns present the planned dose, the film dose, and the gamma results at 3%, 0.3 mm and 7%, 0.3 mm, respectively. Each image has dimensions of 20 × 20 mm.

Figure 6a illustrates the results of the gamma analyses for one film from each centre for the static plans. Three key observations may be made:

1. For several centres (e.g. S1, S2, S5 and S6), the measured dose outside the field was notably higher than the planned dose, as can be seen by the red regions surrounding the field in the gamma images.
2. The largest discrepancy between the planned and delivered dose distributions is in the corners of the square fields, shown by the blue regions in the corner of each field in the gamma images for centres S1–S5. In the measured fields the corners have a more rounded profile and a lower dose than predicted by the TPS.
3. At centre S6, the 10 × 10 mm^2^ field was in fact modelled by a 10 mm diameter circular field. This was a deliberate choice by that centre to minimise the number of apertures that had to be commissioned. As noted above, the corners of the square field measured in the film disagreed with those modelled by the TPS, therefore the use of a circular model is not as unrealistic as might be expected.

Figure 6b illustrates the results of the gamma analyses for one film from each centre for the arc plans.

**Figure 7:**
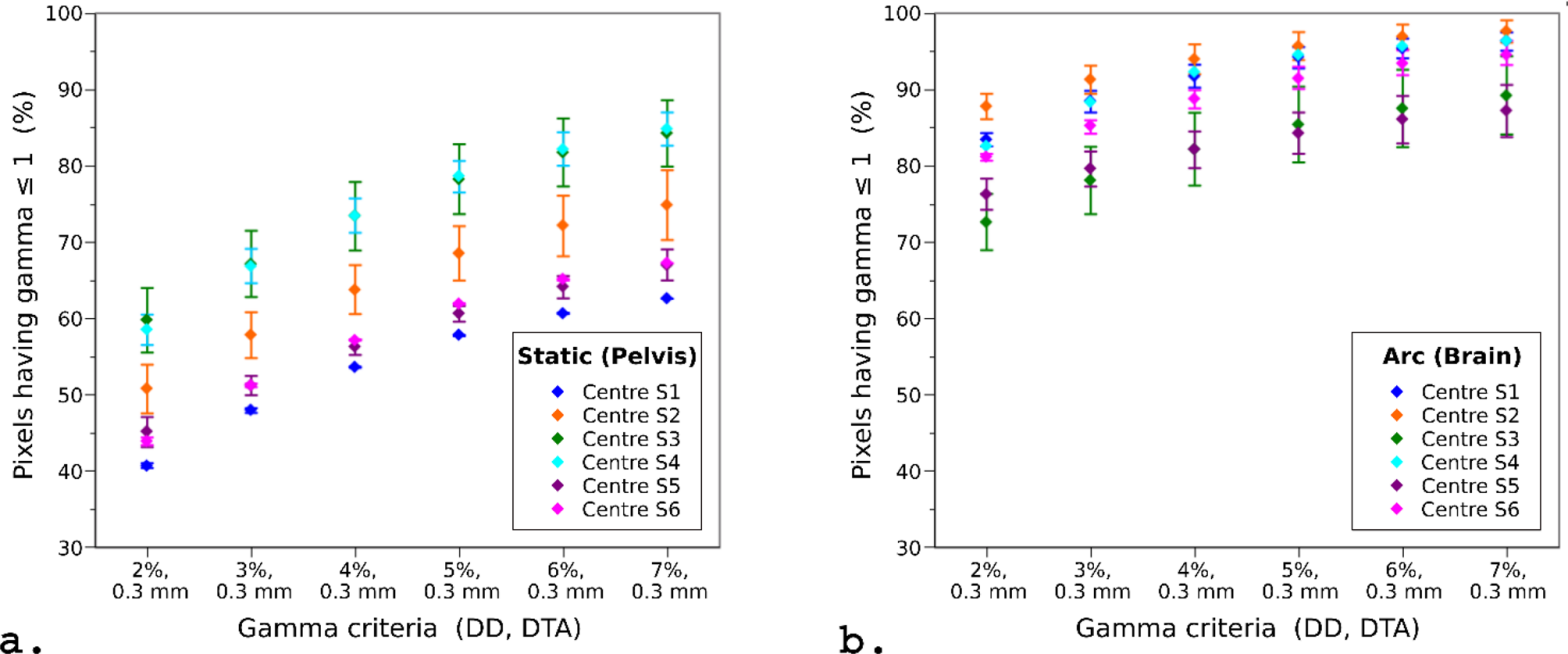
Results of the gamma analyses for all films from each centre for the (**a**) static beam (pelvis) and (**b**) arc (brain) plans. The number of static and arc films analysed for each centre were 2,2,2,2,2,2 and 2,2,1,2,2,3, respectively. The points represent the mean, and the error bars represent the range of results.

The results for all gamma analyses for the static and arc plans are summarised in Fig. 7. Although the 10 × 10 mm^2^ static plans were simpler than the arc plans, the static films consistently had a smaller proportion of passing pixels (γ ≤ 1) mainly because the results were dominated by the discrepancies at the corners of the field and in the out-of-field region. In contrast, in the arc plans the steeper out-of-plane dose gradient tended to result in a higher proportion of passing pixels. Results for all centres were broadly comparable in terms of the trend of the gamma pass rate as a function of the dose difference (DD) criterion.

A summary of the EBT3 dosimetry results can be found in Supplementary Table S4.

### Comparison of alanine and Gafchromic EBT3 film measurements of absolute dose

**Figure 8:**
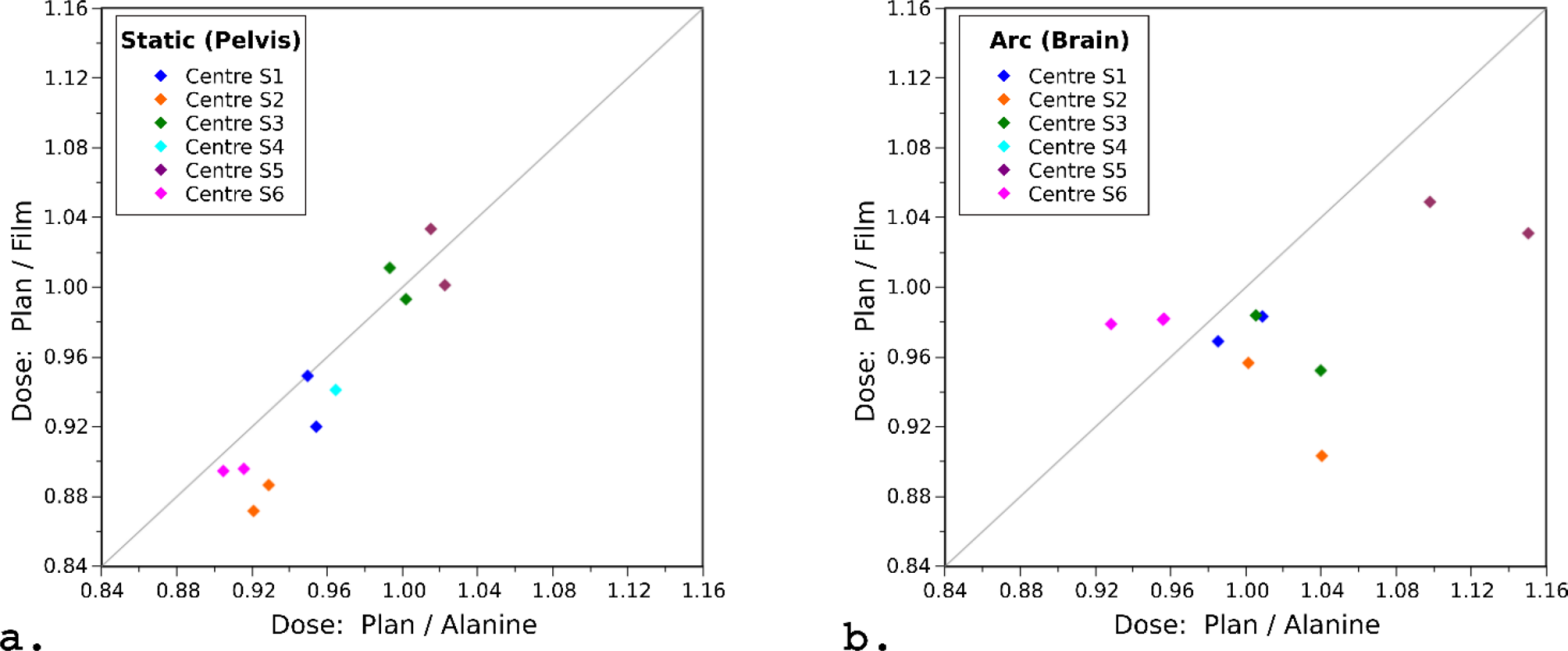
Comparison of the absolute dose measurements acquired using alanine pellets and Gafchromic EBT3 film for (**a**) the static 10 × 10 mm fields and (**b**) the arc deliveries. Results are presented in terms of the ratio of the planned dose to the measured dose (i.e. TPS/Film and TPS/Alanine). A ratio > 1 indicates that the planned dose was greater than the measured dose. The diagonal line indicates the line of agreement between the alanine and film measurements.

Figure 8 compares the absolute dose measurements obtained using Gafchromic EBT3 film and alanine pellets. Data is only shown for matched film and alanine measurements: i.e. where the film and alanine were irradiated together on a single irradiation to allow for a fair comparison of EBT3 and alanine results. No matched film and alanine measurements were available for the arc plan at centre S4 due to the alanine pellets being damaged following irradiation and prior to read-out. The data in Fig. 8 is presented in terms of the ratio of the planned dose to the measured dose, showing results for alanine and EBT3 on the x and y axes, respectively. The diagonal line indicates the line of agreement between alanine and EBT3. For data lying to the upper-left of the diagonal line the EBT3 measurement was lower than alanine, while for data lying to the lower-right of the diagonal line the EBT3 measurement was greater than alanine.

Figure 8a compares the EBT3 and alanine measurements of dose for the static irradiations. The data lie close to the diagonal, indicating that EBT3 and alanine were generally in good agreement. However, the film measurements were typically higher than alanine (by 1.6 ± 2.2%, mean ± standard deviation), and therefore the data are found mainly to the lower-right of the diagonal.

Figure 8b compares the EBT3 and alanine measurements of dose for the arc irradiations. The agreement between film and alanine was worse than for the static plans, which was likely due to the increased complexity of the arc plans, with steeper dose gradients and a high dose region no larger than the dimensions of the alanine pellet. Again the film measurement was typically greater than the alanine, although with a larger variation (2.9 ± 5.6%, mean ± standard deviation) than for the static plans.

### Practical problems encountered during the audit

During the audit several problems were encountered:

1. One of the phantoms had warped during printing making the alanine cavities smaller than designed, and this was not detected during phantom quality control. This meant several pellets were damaged upon removal and were therefore unreadable (arc data for S4). The poor fit of the alanine pellet in the warped phantom also resulted in an air gap beneath the film. This resulted in an artefact in the planned dose distribution adjacent to and downstream of the film. To prevent this affecting the gamma analyses, the planned dose in the air gap was corrected using interpolation. Although PLA is more robust than other commonly used 3D printing materials, such as acrylonitrile butadiene styrene^25^, for future phantom designs the size of the pellet cavity should be reviewed to ensure both potential warping of the phantom and variation in the dimensions of individual alanine pellets are taken into account.
2. One centre mislabelled the plan information upon return so these had to be manually matched to the films during analysis.
3. One centre (S4) mis-interpreted the planning guidance and used an arc in the transverse plane rather than one in the sagittal plane. However, for the purpose of the audit the use of a transverse arc was also acceptable, since the aim was to test agreement between the planned and delivered dose. However, this demonstrates the need for unambiguous guidance in multi-institutional studies.
4. One centre inserted the film the wrong way in the phantom, but due to the symmetrical structure of EBT3 film this had no impact on the analysis.
5. Finally, one centre sent incomplete dose information and the original plan was removed from their TPS. However, it was possible to re-create the plan within Muriplan using the data that had been exported, allowing the dose to be recalculated.

## Discussion

We present results from a preclinical dosimetry audit of Xstrahl SARRP systems at 6 institutions in the UK using a realistic murine phantom. The results of the questionnaire suggest that without a routine protocol there is still some way to go before a consensus is reached across the country. One of the contributing factors to a paucity of rigorous dosimetry protocols is insufficient dosimetry knowledge or support from clinical physics colleagues^4^. Some centres rely on the manufacturer to complete the dosimetry checks, which has the advantage that the checks are done in a consistent fashion by staff who are expertly trained on the system. However, this is not a guarantee that the irradiator has been properly calibrated^37^, and further checks should be performed by appropriate personnel for validation. The responses to the questionnaire also indicate that there may be a lack of support at the institutional level, and the lack of independence in the QA process increases the risk of a systemic problem going unnoticed^37^. Also of importance is the wide variation in the frequency of output checks (bi-monthly to annually) and the traceability of the chambers used for those checks. These should be addressed as the output of a SARRP system can potentially drift over time, with Feddersen et al*.*^10^ reporting a decline of almost 4% over an 18 month period. There did not appear to be any correlation between the frequency and traceability of the QA checks performed and the accuracy of the dosimetry within the present study.

Only one centre reported most of the suggested requirements for accurate reporting recommended by Verhaegen et al*.*^18^. Similarly, many peer-reviewed articles fail to report basic details that are necessary to allow a study to be reproduced or compared to other studies^7,19^. Incomplete reporting of these experimental parameters adds to the biological sources of error that are complex and poorly understood, and is often attributed to insufficient physics expertise among users^19^.

One simple proposed standardized QA methodology is to make use of the in-built electronic portal imaging device, which has shown to be a stable and convenient tool to assess beam quality, energy, output, profile and targeting and verify delivered doses^38,39^. A thorough QA procedure would include the use of ionisation chambers for calibration (with reference to a primary dosimetry standard), film for 2D measurements and a smaller detector to validate dose at submillimetre resolution^40,41^, such as MOSFETs (metal–oxide semiconductor field-effect transistors) or TLDs (thermoluminescent detectors)^4^. However to implement this the issue of physics expertise must first be addressed. Currently, most centres included in this audit use ionisation chambers for QA. Only two centres also use film as part of their QA process.

When the SARRP was first developed over a decade ago the suggested in vivo dosimetry tolerance was 5–10%^2^, consistent with the then 5% target used by audits of clinical low or medium energy X-ray irradiators, with action points if results exceed 10%^42^. This was especially important since it is documented that a 10% dose difference can lead to mortality rates in some mice strains of up to 90%^43^. Apart from 1 delivery from centre S6, the static field deliveries were all within 10% of the planned dose as measured using the alanine dosimeters, with the majority being < 5% (see Fig. 5a). The delivery which failed to meet the 10% tolerance was marginal (being 10.5% high). There are several uncertainties that may contribute to these discrepancies. The accuracy of the HVL measurement and the calculation of the correction factors for the alanine energy dependence (estimated to be 4.8%) or the difference in beam quality between the reference beam at NPL (^60^Co) and the SARRPs (X-ray) used^23^. Additional uncertainties in the applied correction factors may come from the difference in the spectra between the SARRPs and the NPL’s reference beam^23^. Using the nominal HVL thickness of 0.67 mm Cu instead of 0.847 mm Cu, which is due to additional beam gating equipment, increases the dose difference by 2.4%. Furthermore, the signal readout has been known to degrade over time, especially in humid environments^44^. However, here the maximum time between irradiation and readout being < 2 months and the pellets being stored in two sealed envelopes the signal should have remained stable^44^. There may also be contributing uncertainties that are related to the TPS calculations such as segmentation thresholds, commissioning or targeting, which are out of the scope of this investigation. It was assumed the CBCT dose was negligible (< 0.85 cGy) in line with other studies^2,45^, and did not contribute to the delivered dose difference. Apart from 1 delivery from centre S5, the alanine measurements were also all within 10% of the planned dose for the arc deliveries (see Fig. 5a). That the measured doses were lower than for the static beams could be due to the width of the arc field and pellet diameter being the same. A small error in the isocentre targeting, either from user or TPS inaccuracies, would result in incomplete coverage of the alanine dosimeter and therefore the average dose measured over its volume would be reduced. There are additional uncertainties due to the rotation of the bed (during the CBCT acquisition) and gantry (during radiation delivery), which may contribute to the overall targeting uncertainty.

Gafchromic EBT3 film is well established as a tool for absolute dosimetry from 0.01 to 30 Gy^30^. The use of the red colour channel alone allows for accurate dosimetry up to 8 Gy, while the use of multiple colour channels extends the range and reduces the uncertainty in the measured dose^46^. In this study we used the red and green channels for dosimetry up to 15 Gy.

Film provides the ability to assess certain features of the delivered dose distribution that cannot be evaluated using point dosimeters such as alanine pellets: the shape of the dose distribution in 2D; the position of the delivered dose; and the dose deposited outside the high dose region. Each of these types of error has the potential to lead to inaccurate conclusions being drawn from in vivo experiments^47^. Increased focus on validating these aspects of the delivered dose distribution, rather than focussing only on the absolute dose delivered to the target region, would help to refine experiments in several ways: better agreement between planned and delivered dose distributions would increase confidence in delivered doses, consequently reducing the number of animals required, and improving control of the delivered dose away from the target may help to reduce the radiation-induced side effects experienced by the animals.

The results illustrated in Fig. 6a show that agreement between the plan and EBT3 film measurements was generally poor at the field corners, where the film measurements showed a more rounded profile and lower dose than predicted by the TPS, and in the out-of-field regions, where the film measurements were notably higher than the planned dose. Dose errors in these regions could be a concern for experiments where the dose to normal tissues in close proximity to the target is important. These issues were observed in all 10 × 10 cm^2^ static field measurements for all centres, indicating that they are not due to a delivery error. Further investigation is warranted into the exact cause of these discrepancies. Film dosimetry is least reliable at low dose levels, and therefore the out-of-field dose discrepancy could potentially be explained by the limitations of film dosimetry. This is less likely for the discrepancies seen at the corners of the field, which are not in a low dose region and spatially are well within the resolution limits of film dosimetry. Previous studies have suggested that the superposition-convolution dose calculation algorithm used in Muriplan does not accurately model the penumbra^9,39^. Implementation and evaluation of alternative dose calculation algorithms, such as using a Monte-Carlo approach, is worthy of investigation since they may have different behaviour in the out-of-field regions. Other parts of the planning and delivery process may also benefit from investigation, such as the use of bulk density overrides to segment tissue types within the CT image. It is worth noting that similar discrepancies were not observed in the arc dose distributions (Fig. 6b), perhaps because of the use of a smaller field size, and because the relative motion of the beam tends to soften field edges parallel to the axis of rotation.

In relation to the use of Gafchromic EBT3 film for measurement of absolute dose, it should be noted that our calibration films were irradiated using a 300 kVp beam, while the SARRP systems tested within the audit used a 220 kVp beam. The response of EBT3 film is known to be sensitive to energy when used for kilovoltage beams^46^, although the formulation of the active layer in the film has been designed to minimise this^48^. Results reported by Bekerat et al.^48^ show that the energy dependence is small at energies above 70 keV, and within the range 220–300 keV is likely to be within a few percent at most. Nevertheless, this has the potential to introduce a small systematic error to the EBT3 absolute dose measurements within the audit.

It was observed that the EBT3 measurements of dose were typically higher than alanine, by 1.6 ± 2.2% for the static plans and 2.9 ± 5.6% for the arc plans. It is worth highlighting that this difference was greater for the arc plans, which is consistent with our earlier remark that the alanine dosimeters may not receive the full planned dose in the event of a small set-up error, due to the width of the arc field and pellet diameter being the same. However, we found no relationship between the dose difference and the residual positional shifts obtained during the film analysis procedure.

The density of soft tissues typically ranges from 0.95 g/cm^3^ (for adipose tissue) to 1.05 g/cm^3^ (for muscle)^49,50^. Furthermore, the phantom used during the Muriplan TPS commissioning is kV-equivalent solid water^11^. Keeping in line with published dosimetry protocols, dosimetry phantoms should be a density close to water (1 g/cm^3^) such that the measurements obtained are within a few percent^51,52^.The ICRU report 44 states corrections factors may be required for absorbed dose measurements obtained with phantoms that introduce uncertainties greater than 1%^50^. The density of the phantoms used in the audit was 1.19 g/cm^3^. This difference potentially impacts on the accuracy of the dose calculation. Within the TPS, the tissue segmentation allows voxels within an image to be assigned as one of 5 discrete materials (air, lung, fat, tissue or bone) whose densities are defined according to ICRU report 44^50,53^. An underestimate in the density of the material defined as ‘tissue’ will lead to the TPS underestimating the attenuation of the beam within the phantom, which we estimate could lead to an error of 1–2% in the calculated dose at the film or alanine detectors. The magnitude of the dose error will be dependent on the field size, the geometry of the phantom, the depth of the dosimeter, and the SSD. This may contribute to the behaviour observed in Fig. 5, where the dose differences between measurement (both alanine and film) and plan are consistently higher for the static plans than for the arc plans. In terms of the variation between institutions any dose error due to the density of the phantom would be systematic, having the same impact on all centres. For future phantom or audit work the choice and density of material used for the phantom should be carefully specified and checked to improve the accuracy of absolute dose measurements.

Finally, many of the practical issues encountered during the audit arose due to the logistics of it being a postal audit as opposed to having being undertaken by the authors. However, a benefit of a postal audit is that it provides information regarding the real use of the machines by the end users. With the exception of the phantom warping, the issues had no real impact on the results but highlight the need for clear instruction for future postal audits.

## Conclusions

Regular end-to-end dosimetry audits complement the QA performed by the user, testing all stages of the planning and delivery process, and provide confirmation that centres’ practices and results are consistent with the wider community. This audit shows the potential of using realistic phantom geometries for evaluation of dose distributions that are representative of experimental scenarios. The use of two different types of dosimeter (film and alanine) allows different features of the dose distributions to be evaluated, and also provides the means to check consistency between the different dosimeters, in this case in terms of absolute dose. This preclinical dosimetry audit found the delivered doses for the simple static field plans to the pelvis to be within 10% of the planned dose to the isocentre for all but one of the measurements across the six centres, but with an overall range of 12.3% between the lowest and highest measured dose, and an overall mean absolute difference of 4.6%. For the more challenging arc plans to the brain all but one measurement was within 10% of the planned dose and the overall mean absolute difference was again 4.6%, but the overall range between the lowest and highest measured dose was larger, at 21.4%. Standardisation of dosimetry protocols would be desirable to improve the agreement between centres in order to prevent such dose differences leading to significantly different biological responses in in vivo experiments. We recommend that phantoms such as the one reported in this study be adopted into routine dosimetry QA protocols. Consistent and regular checks will ensure accurate and precise dosimetry and improve the reproducibility of research results at different institutions.

## Supplementary Information

Supplementary Information.

## Data Availability

All data generated or analysed during this study are included in this published article (and its Supplementary Information files), or are available from the corresponding author on reasonable request.
