# Supplementary material for: A preclinical radiotherapy dosimetry audit using a realistic 3D printed murine phantom
Source: Sci Rep. 2022 Apr 26;12:6826. doi: 10.1038/s41598-022-10895-5 (PMC9042835; doi:10.1038/s41598-022-10895-5)
Supplement: Supplementary file 1 — Supplementary Information. [file 41598_2022_10895_MOESM1_ESM.pdf]

A preclinical radiotherapy dosimetry audit using a realistic 3D printed murine phantom

Emma R Biglin[1], Adam H Aitkenhead[1,2], Gareth J Price[1,3], Amy L Chadwick[1,3], Elham Santina[1,3], Kaye J Williams[4], Karen J Kirkby[1,3].

[1]*Division of Cancer Sciences, Faculty of Biology, Medicine and Health, The University of Manchester, Manchester, UK.*
[2]*Christie Medical Physics and Engineering, The Christie NHS Foundation Trust, Manchester, UK.*
[3]*The Christie NHS Foundation Trust, Manchester, UK.*
[4]*Division of Pharmacy and Optometry, Faculty of Biology, Medicine and Health, University of Manchester, Manchester, UK*.

Supplementary Table S1
The audit protocol each institution was instructed to follow.

Audit aims	Investigate the current status of pre-clinical dosimetry of SARRPs in the UK.
Equipment provided	• Two mice phantoms<br>• Gafchromic EBT3 film<br>o Please handle by the edges<br>• Alanine pellets<br>o Please handle carefully with gloves and avoid contact with water<br>• USB stick<br>o Please save the dose information (.nrrd file), treatment plan (CurrentRecon.nrrd) and SARRP CBCT.
Pre-irradiation checks	Prior to irradiating please perform any standard QA checks and record the current room temperature and the date/time of irradiations:
Irradiation: Static beam - Pelvis	1. Insert an alanine pellet in the pelvis cavity and place the film labelled “A1” on top and clip the two halves of the phantom together<br>2. Place the phantom on the bed in the prone position, head facing the gantry<br>a. Use the lasers to ensure the film is parallel to the floor and not rotated<br>3. CBCT scan the phantom<br>a. Standard SARRP imaging settings: kV, mA<br>4. Define segmentation thresholds for air & soft tissue<br>a. Air: , Soft Tissue:<br>5. Set the isocentre as the centre of the alanine pellet<br>6. Irradiate<br>a. 10 mm x 10 mm collimator<br>b. Gantry at 0°<br>c. Bed rotation at 0°<br>d. 12 Gy<br>e. Standard SARRP irradiation settings: kV, mA<br>7. Place the alanine pellet back in the pre-labelled plastic envelope and record this as measurement A1. Place the film in the white envelope provided.<br>8. Repeat this measurement using another alanine pellet and the film marked “A2”.
Irradiation: Arc beam - Brain	1. Insert an alanine pellet in the brain cavity and place the film labelled “B1” on top and clip the two halves of the phantom together<br>2. Place the phantom on the bed in the prone position, head to gantry<br>a. Use the lasers to ensure the film is parallel to the floor and not rotated<br>b. CBCT scan the phantom using the standard SARRP imaging settings<br>3. Defined threshold for air & soft tissue<br>4. Set the isocentre as the centre of the alanine pellet<br>5. Irradiate using the standard irradiation settings:<br>a. 5 mm x 5 mm collimator<br>b. Gantry to rotate from -45° to 45°<br>c. Bed rotation at 90°<br>d. 12 Gy<br>6. Place the alanine pellet back in the pre-labelled plastic envelope and record this as measurement B1. Place the film in the white envelope provided.<br>7. Repeat this measurement using another alanine pellet and the film marked “B2”.

Supplementary Table S2

Summary of the questionnaire results from each institution.

Blank spaces indicate where an answer was not provided.

Centre	What should the dose tolerance be?	Use of irradiator and techniques used	Who performs QA checks? How often? What equipment is used?	Is the equipment calibrated against a primary standard?	How often is the output checked?	How often is the output calibrated? When was this last?	What irradiation parameters should be reported?	What dosimetry parameters should be reported?
S1	5%	Cell culture & animals Static & arc	Physicists. Farmer chamber type 30010 and film	Y	Yearly			
S2	5%	Cell culture & animals Static	Dedicated user & physicists. Every two months or after service. PTW Unidose & Farmer chamber type 30012	Y	Bi-monthly	2018	Device used, gating, dose delivered, dose rate, fractionation, image guidance, irradiation technique, field size, SSD, backscatter, couch, D95	HVL, voltage, filtration, dosimetry protocol (air or water), output measurements, SSD, depth, backscatter, medium, calibration conditions.
S3		Animals Static	Dedicated user. Before every use. Unidose	Calibrated by PTW				
S4	5%	Animals Arc	Manufacturer & dedicated user. Every 6 months		Every 6 months	Every 6 months. 2019	Correct dose and isocentre	Isolines dosages giving less or avoiding oral cavity
S5		Cell culture Arc	Manufacturer. Every 6 months. Isocentre and machine colorimetric system				If the area that has to be irradiated is receiving the correct dose	Consistency of dosing
S6	10-15%	Animals Static	Dedicated user. Daily/weekly geometry checks. Farmer chamber & film	Y	Yearly	Every 2 years. 2016	Dose, dose rate, irradiation protocol & geometry and collimation	Commissioning, tube current and filtration, D90
S7	5%	Cell culture & animals Static	All users. Daily (alignment)/ monthly (shielding & dosimetry). PTW, semiflex, farmer	Calibrated by PTW	Monthly	When necessary. 2019	Filters, setup, energy, mAs	Chamber, field size, beam quality

Supplementary Table S3

Full data set for all alanine measurements and percentage difference compared to the treatment planning system (TPS) calculated dose. The pairing label links to the data in table S4, indicating film and alanine data that were acquired during a single irradiation.

($R_{Q,Q0}$: Q - kVp X-ray quality, Q0 - Co-60 quality)

Institution	Scenario	Pairing label	TPS dose (Gy)	Alanine result (Gy)	HVL (mm Cu)	$R_{Q,Q0}$	Alanine result corrected (Gy)	Difference between TPS and corrected alanine (%)
S1	Static	a	12.096	10.073	0.670	0.793	12.696	5.3
	Static	b	12.062	9.976			12.574	4.7
	Arc	c	12.041	9.609			12.111	1.4
	Arc	d	11.988	9.379			11.821	−0.9
S2	Static	e	12.070	10.578	0.847	0.813	13.014	8.5
	Static	f	12.067	10.517			12.939	7.6
	Arc	g	12.093	9.745			11.989	−0.2
	Arc	h	12.137	9.404			11.569	−3.9
S3	Static	i	12.084	9.523	0.670	0.793	12.003	−0.2
	Static	j	12.117	9.509			11.985	0.6
	Arc	k	12.114	9.164			11.550	−3.8
	Arc	l	12.049	9.458			11.921	−0.6
S4	Static	-	12.117	9.883	0.670	0.793	12.457	3.1
	Static	m	12.173	9.971			12.567	3.6
S5	Static	n	11.860	9.283	0.700	0.797	11.647	−1.5
	Static	o	11.953	9.237			11.589	−2.3
	Arc	p	11.952	8.604			10.795	−9.0
	Arc	q	12.036	8.287			10.397	−13.1
S6	Static	r	12.089	10.414	0.650	0.791	13.167	9.2
	Static	s	11.928	10.392			13.140	10.5
	Arc	t	11.988	10.088			12.755	7.7
	Arc	u	12.065	9.879			12.491	4.5
	Arc	v	11.993	9.839			12.440	4.6

Supplementary Table S4

Full data set for all film measurements compared to the treatment planning system (TPS) calculated dose. The pairing label links to the data in table S3, indicating film and alanine data that were acquired during a single irradiation.

Institution	Scenario	Pairing label	Residual positional correction (mm)	Normalisation factor (TPS/Film)	Gamma analysis: Percentage of pixels where γ ≤ 1					
					DD: 2% DTA: 0.3 mm	DD: 3% DTA: 0.3 mm	DD: 4% DTA: 0.3 mm	DD: 5% DTA: 0.3 mm	DD: 6% DTA: 0.3 mm	DD: 7% DTA: 0.3 mm
S1	Static	a	0.78	0.949	40.4	47.7	53.6	57.9	60.8	62.6
	Static	b	0.71	0.920	41.1	48.3	53.7	57.7	60.6	62.6
	Arc	c	1.06	0.969	82.6	87.0	90.3	92.8	94.1	95.1
	Arc	d	1.12	0.983	84.3	89.9	93.3	95.6	96.7	97.5
S2	Static	e	0.63	0.872	54.0	60.9	67.0	72.1	76.1	79.5
	Static	f	0.28	0.886	47.6	54.8	60.6	65.0	68.2	70.3
	Arc	g	1.22	0.957	86.2	89.5	92.0	93.9	95.3	96.2
	Arc	h	0.36	0.903	89.5	93.2	96.0	97.6	98.6	99.1
S3	Static	i	0.57	0.993	55.6	62.9	68.9	73.7	77.3	79.9
	Static	j	0.32	1.011	64.0	71.5	77.9	82.9	86.3	88.6
	Arc	k	0.00	0.952	69.0	73.7	77.5	80.5	82.5	84.2
	Arc	l	0.51	0.984	76.4	82.5	87.0	90.4	92.6	94.4
S4	Static	-	0.45	0.920	56.5	64.7	71.3	76.5	80.0	82.7
	Static	m	0.38	0.941	60.5	69.2	75.8	80.7	84.4	87.0
	Arc	-	0.85	1.109	82.7	88.3	92.3	94.5	95.7	96.4
S5	Static	n	0.82	1.033	43.2	50.0	55.3	59.6	62.7	65.0
	Static	o	1.41	1.001	47.1	52.5	57.3	61.7	65.6	69.1
	Arc	p	0.95	1.049	78.4	81.9	84.5	87.0	89.2	90.7
	Arc	q	1.16	1.031	74.3	77.3	79.7	81.6	83.0	83.8
S6	Static	r	0.59	0.896	43.4	51.0	57.3	62.0	65.2	67.2
	Static	s	1.05	0.894	44.4	51.5	57.1	61.9	65.0	67.3
	Arc	t	0.43	0.979	81.3	86.0	90.0	93.0	95.2	96.5
	Arc	u	0.42	0.982	80.7	84.3	87.6	90.1	91.9	93.3
	Arc	v	0.50	0.981	81.6	85.5	88.8	91.3	93.0	94.2
